# 4-Allyl-2-meth­oxy­phenyl 2-acet­oxy­benzoate

**DOI:** 10.1107/S1600536811021416

**Published:** 2011-06-11

**Authors:** Xi-Wang Liu, Jian-Yong Li, Ya-Jun Yang, Ji-Yu Zhang

**Affiliations:** a Key Laboratory of New Animal Drug Project, Gansu Province Key Laboratory of Veterinary Drug Discovery, Ministry of Agriculture, Lanzhou Institute of Animal Science and Veterinary Pharmaceutics, Chinese Academy of Agricultural Sciences, Lanzhou 730050, People’s Republic of China

## Abstract

In the title compound, C_19_H_18_O_5_, the ester group is twisted with respect to the acetyl­salicylic acid and eugenol rings at dihedral angles of 22.48 (2) and 81.07 (1)°, respectively. The dihedral angle between the two benzene rings is 60.72 (1)°. The crystal packing exhibits no significantly short inter­molecular contacts.

## Related literature

For background regarding the medicinal properties of eugenol, see: Feng & Lipton (1987 ▶); Dohi *et al.* (1989 ▶). For the synthesis of the aspirin eugenol ester and its biological activity, see: Li *et al.* (2011 ▶). 
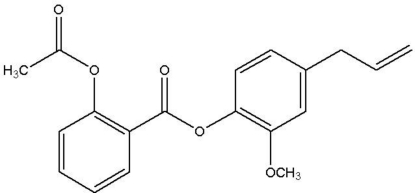


## Experimental

### 

#### Crystal data

C_19_H_18_O_5_
*M* *_r_* = 326.33
Monoclinic, 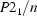

*a* = 10.60 (2) Å
*b* = 12.58 (2) Å
*c* = 13.23 (2) Å
β = 109.020 (17)°
*V* = 1667 (5) Å^3^
*Z* = 4
Mo *K*α radiation
μ = 0.09 mm^−1^
*T* = 296 K
0.26 × 0.24 × 0.22 mm

#### Data collection

Bruker APEXII CCD diffractometer
Absorption correction: multi-scan (*SADABS*; Sheldrick, 1996 ▶) *T*_min_ = 0.976, *T*_max_ = 0.980
8695 measured reflections
3089 independent reflections
1786 reflections with *I* > 2σ(*I*)
*R*_int_ = 0.054

#### Refinement

*R*[*F* ^2^ > 2σ(*F* ^2^)] = 0.051
*wR*(*F* ^2^) = 0.142
*S* = 1.01
3089 reflections
220 parameters
H-atom parameters constrained
Δρ_max_ = 0.49 e Å^−3^
Δρ_min_ = −0.25 e Å^−3^

### 

Data collection: *APEX2* (Bruker, 2007 ▶); cell refinement: *SAINT* (Bruker, 2007 ▶); data reduction: *SAINT*; program(s) used to solve structure: *SHELXS97* (Sheldrick, 2008 ▶); program(s) used to refine structure: *SHELXL97* (Sheldrick, 2008 ▶); molecular graphics: *SHELXTL* (Sheldrick, 2008 ▶); software used to prepare material for publication: *SHELXTL*.

## Supplementary Material

Crystal structure: contains datablock(s) I, global. DOI: 10.1107/S1600536811021416/ez2244sup1.cif

Structure factors: contains datablock(s) I. DOI: 10.1107/S1600536811021416/ez2244Isup2.hkl

Supplementary material file. DOI: 10.1107/S1600536811021416/ez2244Isup3.cml

Additional supplementary materials:  crystallographic information; 3D view; checkCIF report

## Figures and Tables

**Fig. 1.**
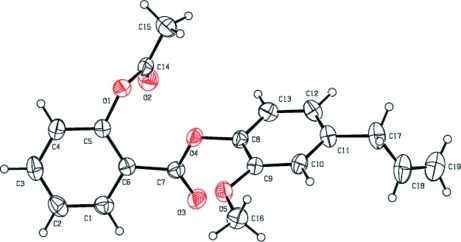
The molecular structure of (I), with atom labels and displacement ellipsoids drawn at the 30% probability level.

## supplementary crystallographic information

### Comment

Aspirin has been widely used as an analgesic and anti-inflammatory drug. As the major constituent of clove oil, eugenol also shows antipyretic activity (Feng & Lipton, 1987) and anti-inflammatory activity (Dohi *et al.*, 1989). In this paper, we report the structure of the title compound, which was synthesized from the reaction of aspirin and eugenol in sodium hydroxide solution.

### Experimental

The title compound was obtained according to the literature method (Li *et al.*, 2011). Acetylsalicylic acid (0.025 mol) and thionyl chloride (2.5 ml) were mixed in 10 ml tetrahydrofuran (THF), and refluxed at 343 K for 2 h. The surplus thionyl chloride and THF were removed under reduced pressure. The target *O*-Acetylsalicylyl chloride was dissolved in 5 ml THF, added dropwise to an iced solution of eugenol (0.025 mol) and sodium hydroxide (0.04 mol) in 40 ml water. After stirring at room temprature for 3 h, the crude product was obtained by filtration. The crystals were obtained by recrystalization from methanol. Elemental analysis: calculated for C_19_ H_18_O_5_: C 69.93%, H 5.56%, O 24.51%; found: C 69.93%, H 5.53%, O 24.54%.

### Refinement

The positions of all H atoms were determined geometrically and refined using a riding model with C—H = 0.93–0.97 Å and *U*_iso_(methyl H) = 1.5*U*_eq_(C) and 1.2*U*_eq_ for other H atoms.

### Crystal data

**Table:**

C_19_H_18_O_5_	*F*(000) = 688
*M**_r_* = 326.33	*D*_x_ = 1.300 Mg m^−^^3^
Monoclinic, *P*2_1_/*n*	Melting point = 344–345 K
Hall symbol: P 21/n	Mo *K*α radiation, λ = 0.71073 Å
*a* = 10.60 (2) Å	Cell parameters from 2334 reflections
*b* = 12.58 (2) Å	θ = 2.3–23.9°
*c* = 13.23 (2) Å	µ = 0.09 mm^−^^1^
β = 109.020 (17)°	*T* = 296 K
*V* = 1667 (5) Å^3^	Block, colorless
*Z* = 4	0.26 × 0.24 × 0.22 mm

### Data collection

**Table:**

Bruker APEXII CCD diffractometer	3089 independent reflections
Radiation source: fine-focus sealed tube	1786 reflections with *I* > 2σ(*I*)
graphite	*R*_int_ = 0.054
φ and ω scans	θ_max_ = 25.5°, θ_min_ = 2.3°
Absorption correction: multi-scan (*SADABS*; Sheldrick, 1996)	*h* = −11→12
*T*_min_ = 0.976, *T*_max_ = 0.980	*k* = −15→15
8695 measured reflections	*l* = −16→15

### Refinement

**Table:**

Refinement on *F*^2^	Secondary atom site location: difference Fourier map
Least-squares matrix: full	Hydrogen site location: inferred from neighbouring sites
*R*[*F*^2^ > 2σ(*F*^2^)] = 0.051	H-atom parameters constrained
*wR*(*F*^2^) = 0.142	*w* = 1/[σ^2^(*F*_o_^2^) + (0.0532*P*)^2^ + 0.6544*P*] where *P* = (*F*_o_^2^ + 2*F*_c_^2^)/3
*S* = 1.01	(Δ/σ)_max_ < 0.001
3089 reflections	Δρ_max_ = 0.49 e Å^−^^3^
220 parameters	Δρ_min_ = −0.25 e Å^−^^3^
0 restraints	Extinction correction: *SHELXL97* (Sheldrick, 2008), Fc^*^=kFc[1+0.001xFc^2^λ^3^/sin(2θ)]^-1/4^
Primary atom site location: structure-invariant direct methods	Extinction coefficient: 0.044 (3)

### Special details

**Table:**

Geometry. All e.s.d.'s (except the e.s.d. in the dihedral angle between two l.s. planes) are estimated using the full covariance matrix. The cell e.s.d.'s are taken into account individually in the estimation of e.s.d.'s in distances, angles and torsion angles; correlations between e.s.d.'s in cell parameters are only used when they are defined by crystal symmetry. An approximate (isotropic) treatment of cell e.s.d.'s is used for estimating e.s.d.'s involving l.s. planes.
Refinement. Refinement of *F*^2^ against ALL reflections. The weighted *R*-factor *wR* and goodness of fit *S* are based on *F*^2^, conventional *R*-factors *R* are based on *F*, with *F* set to zero for negative *F*^2^. The threshold expression of *F*^2^ > σ(*F*^2^) is used only for calculating *R*-factors(gt) *etc*. and is not relevant to the choice of reflections for refinement. *R*-factors based on *F*^2^ are statistically about twice as large as those based on *F*, and *R*- factors based on ALL data will be even larger.

### Fractional atomic coordinates and isotropic or equivalent isotropic displacement parameters (Å^2^)

**Table:**

	*x*	*y*	*z*	*U*_iso_*/*U*_eq_	
C1	0.5429 (3)	0.8815 (2)	1.1109 (2)	0.0552 (7)	
H1	0.5154	0.9490	1.0842	0.066*	
C2	0.6740 (3)	0.8662 (3)	1.1758 (3)	0.0651 (9)	
H2	0.7329	0.9233	1.1933	0.078*	
C3	0.7161 (3)	0.7671 (3)	1.2139 (2)	0.0667 (9)	
H3	0.8038	0.7566	1.2575	0.080*	
C4	0.6290 (3)	0.6826 (3)	1.1880 (2)	0.0589 (8)	
H4	0.6582	0.6150	1.2135	0.071*	
C5	0.4980 (2)	0.6979 (2)	1.1242 (2)	0.0454 (6)	
C6	0.4519 (2)	0.7981 (2)	1.0850 (2)	0.0445 (6)	
C7	0.3121 (3)	0.8251 (2)	1.0192 (2)	0.0460 (6)	
C8	0.0856 (2)	0.7755 (2)	0.9839 (2)	0.0457 (7)	
C9	0.0242 (2)	0.8431 (2)	1.0363 (2)	0.0451 (6)	
C10	−0.1134 (3)	0.8549 (2)	0.9951 (2)	0.0522 (7)	
H10	−0.1563	0.8996	1.0295	0.063*	
C11	−0.1880 (3)	0.8015 (2)	0.9038 (2)	0.0563 (7)	
C12	−0.1244 (3)	0.7362 (2)	0.8531 (2)	0.0619 (8)	
H12	−0.1735	0.7007	0.7911	0.074*	
C13	0.0134 (3)	0.7226 (2)	0.8936 (2)	0.0563 (8)	
H13	0.0560	0.6776	0.8593	0.068*	
C14	0.3662 (3)	0.5694 (2)	1.0060 (3)	0.0523 (7)	
C15	0.2725 (3)	0.4813 (3)	1.0018 (3)	0.0784 (10)	
H15A	0.2235	0.4650	0.9286	0.118*	
H15B	0.2114	0.5019	1.0382	0.118*	
H15C	0.3216	0.4197	1.0359	0.118*	
C16	0.0468 (3)	0.9576 (2)	1.1848 (2)	0.0664 (8)	
H16A	−0.0043	1.0124	1.1391	0.100*	
H16B	0.1152	0.9894	1.2436	0.100*	
H16C	−0.0109	0.9156	1.2118	0.100*	
C17	−0.3382 (3)	0.8185 (3)	0.8597 (3)	0.0794 (10)	
H17A	−0.3803	0.7545	0.8231	0.095*	
H17B	−0.3719	0.8312	0.9186	0.095*	
C18	−0.3746 (4)	0.9083 (4)	0.7853 (5)	0.1113 (15)	
H18	−0.3360	0.9723	0.8151	0.134*	
C19	−0.4456 (4)	0.9161 (4)	0.6917 (4)	0.1173 (16)	
H19A	−0.4886	0.8564	0.6548	0.141*	
H19B	−0.4565	0.9817	0.6575	0.141*	
O1	0.41425 (17)	0.60944 (14)	1.10660 (15)	0.0517 (5)	
O4	0.22313 (15)	0.75382 (14)	1.03094 (14)	0.0512 (5)	
O2	0.3986 (2)	0.60219 (17)	0.93385 (16)	0.0684 (6)	
O3	0.28083 (19)	0.90274 (16)	0.96467 (17)	0.0681 (6)	
O5	0.10653 (18)	0.89150 (15)	1.12579 (15)	0.0582 (5)	

### Atomic displacement parameters (Å^2^)

**Table:**

	*U*^11^	*U*^22^	*U*^33^	*U*^12^	*U*^13^	*U*^23^
C1	0.0542 (17)	0.0533 (16)	0.0632 (19)	−0.0034 (13)	0.0263 (16)	−0.0015 (15)
C2	0.0491 (18)	0.076 (2)	0.073 (2)	−0.0145 (16)	0.0245 (17)	−0.0128 (18)
C3	0.0390 (16)	0.095 (3)	0.063 (2)	0.0017 (17)	0.0112 (15)	−0.0091 (19)
C4	0.0483 (17)	0.0676 (19)	0.0568 (19)	0.0101 (15)	0.0115 (15)	0.0017 (16)
C5	0.0426 (15)	0.0516 (16)	0.0420 (15)	0.0016 (12)	0.0139 (13)	−0.0015 (13)
C6	0.0431 (14)	0.0494 (15)	0.0435 (15)	0.0003 (12)	0.0177 (13)	−0.0015 (13)
C7	0.0468 (15)	0.0433 (15)	0.0506 (16)	−0.0001 (12)	0.0197 (13)	0.0004 (14)
C8	0.0382 (14)	0.0477 (15)	0.0518 (17)	0.0038 (12)	0.0153 (13)	0.0055 (13)
C9	0.0424 (15)	0.0473 (14)	0.0446 (16)	0.0017 (12)	0.0127 (13)	0.0014 (13)
C10	0.0426 (15)	0.0616 (17)	0.0552 (18)	0.0108 (13)	0.0196 (14)	0.0042 (15)
C11	0.0407 (15)	0.076 (2)	0.0493 (17)	−0.0026 (14)	0.0110 (14)	0.0006 (17)
C12	0.0541 (18)	0.075 (2)	0.0530 (18)	−0.0113 (15)	0.0126 (15)	−0.0093 (16)
C13	0.0573 (18)	0.0576 (17)	0.0596 (19)	−0.0026 (14)	0.0268 (16)	−0.0067 (15)
C14	0.0411 (15)	0.0520 (16)	0.0586 (19)	0.0105 (12)	0.0090 (15)	−0.0025 (16)
C15	0.064 (2)	0.072 (2)	0.093 (3)	−0.0104 (17)	0.0180 (19)	−0.015 (2)
C16	0.073 (2)	0.071 (2)	0.0570 (19)	0.0082 (17)	0.0236 (17)	−0.0100 (16)
C17	0.0428 (17)	0.115 (3)	0.074 (2)	−0.0002 (19)	0.0107 (17)	0.005 (2)
C18	0.048 (2)	0.131 (4)	0.138 (4)	0.020 (2)	0.009 (3)	0.003 (4)
C19	0.080 (3)	0.128 (4)	0.121 (4)	0.026 (3)	0.001 (3)	0.012 (3)
O1	0.0531 (11)	0.0474 (11)	0.0527 (12)	0.0001 (9)	0.0146 (10)	0.0027 (9)
O4	0.0372 (9)	0.0490 (10)	0.0684 (13)	0.0070 (8)	0.0188 (9)	0.0113 (9)
O2	0.0699 (14)	0.0784 (15)	0.0533 (13)	0.0034 (11)	0.0150 (12)	−0.0029 (11)
O3	0.0577 (12)	0.0600 (13)	0.0808 (15)	0.0010 (10)	0.0144 (11)	0.0244 (12)
O5	0.0482 (11)	0.0671 (13)	0.0543 (12)	0.0069 (10)	0.0097 (10)	−0.0124 (10)

### Geometric parameters (Å, °)

**Table:**

C1—C6	1.390 (4)	C11—C17	1.521 (5)
C1—C2	1.387 (4)	C12—C13	1.392 (5)
C1—H1	0.9300	C12—H12	0.9300
C2—C3	1.364 (5)	C13—H13	0.9300
C2—H2	0.9300	C14—O2	1.189 (4)
C3—C4	1.376 (5)	C14—O1	1.357 (4)
C3—H3	0.9300	C14—C15	1.477 (4)
C4—C5	1.383 (4)	C15—H15A	0.9600
C4—H4	0.9300	C15—H15B	0.9600
C5—C6	1.390 (4)	C15—H15C	0.9600
C5—O1	1.394 (4)	C16—O5	1.423 (3)
C6—C7	1.492 (4)	C16—H16A	0.9600
C7—O3	1.195 (3)	C16—H16B	0.9600
C7—O4	1.346 (3)	C16—H16C	0.9600
C8—C13	1.363 (4)	C17—C18	1.465 (6)
C8—C9	1.386 (4)	C17—H17A	0.9700
C8—O4	1.412 (4)	C17—H17B	0.9700
C9—O5	1.364 (3)	C18—C19	1.226 (6)
C9—C10	1.389 (4)	C18—H18	0.9300
C10—C11	1.382 (4)	C19—H19A	0.9300
C10—H10	0.9300	C19—H19B	0.9300
C11—C12	1.368 (4)		
			
C6—C1—C2	121.5 (3)	C13—C12—H12	119.7
C6—C1—H1	119.3	C8—C13—C12	119.7 (3)
C2—C1—H1	119.3	C8—C13—H13	120.1
C3—C2—C1	119.8 (3)	C12—C13—H13	120.1
C3—C2—H2	120.1	O2—C14—O1	123.0 (3)
C1—C2—H2	120.1	O2—C14—C15	126.7 (3)
C2—C3—C4	120.1 (3)	O1—C14—C15	110.4 (3)
C2—C3—H3	119.9	C14—C15—H15A	109.5
C4—C3—H3	119.9	C14—C15—H15B	109.5
C3—C4—C5	120.2 (3)	H15A—C15—H15B	109.5
C3—C4—H4	119.9	C14—C15—H15C	109.5
C5—C4—H4	119.9	H15A—C15—H15C	109.5
C4—C5—C6	121.0 (3)	H15B—C15—H15C	109.5
C4—C5—O1	116.6 (3)	O5—C16—H16A	109.5
C6—C5—O1	122.3 (3)	O5—C16—H16B	109.5
C1—C6—C5	117.5 (3)	H16A—C16—H16B	109.5
C1—C6—C7	116.7 (3)	O5—C16—H16C	109.5
C5—C6—C7	125.8 (2)	H16A—C16—H16C	109.5
O3—C7—O4	123.2 (3)	H16B—C16—H16C	109.5
O3—C7—C6	124.4 (2)	C18—C17—C11	112.3 (3)
O4—C7—C6	112.4 (2)	C18—C17—H17A	109.1
C13—C8—C9	121.1 (3)	C11—C17—H17A	109.1
C13—C8—O4	119.7 (2)	C18—C17—H17B	109.1
C9—C8—O4	118.9 (3)	C11—C17—H17B	109.1
O5—C9—C8	115.9 (3)	H17A—C17—H17B	107.9
O5—C9—C10	125.9 (2)	C19—C18—C17	132.9 (5)
C8—C9—C10	118.3 (3)	C19—C18—H18	113.5
C11—C10—C9	121.3 (2)	C17—C18—H18	113.5
C11—C10—H10	119.4	C18—C19—H19A	120.0
C9—C10—H10	119.4	C18—C19—H19B	120.0
C12—C11—C10	119.1 (3)	H19A—C19—H19B	120.0
C12—C11—C17	121.2 (3)	C14—O1—C5	118.3 (2)
C10—C11—C17	119.7 (3)	C7—O4—C8	118.8 (2)
C11—C12—C13	120.5 (3)	C9—O5—C16	117.7 (2)
C11—C12—H12	119.7		
			
C6—C1—C2—C3	1.2 (4)	C9—C10—C11—C12	−0.4 (4)
C1—C2—C3—C4	0.1 (4)	C9—C10—C11—C17	−178.6 (3)
C2—C3—C4—C5	−0.7 (4)	C10—C11—C12—C13	0.9 (4)
C3—C4—C5—C6	0.1 (4)	C17—C11—C12—C13	179.1 (3)
C3—C4—C5—O1	−176.5 (2)	C9—C8—C13—C12	−0.1 (4)
C2—C1—C6—C5	−1.7 (4)	O4—C8—C13—C12	173.8 (2)
C2—C1—C6—C7	176.9 (2)	C11—C12—C13—C8	−0.6 (4)
C4—C5—C6—C1	1.0 (4)	C12—C11—C17—C18	−90.0 (5)
O1—C5—C6—C1	177.5 (2)	C10—C11—C17—C18	88.1 (4)
C4—C5—C6—C7	−177.5 (2)	C11—C17—C18—C19	122.2 (5)
O1—C5—C6—C7	−1.0 (4)	O2—C14—O1—C5	4.8 (4)
C1—C6—C7—O3	21.1 (4)	C15—C14—O1—C5	−176.0 (2)
C5—C6—C7—O3	−160.4 (3)	C4—C5—O1—C14	−113.6 (3)
C1—C6—C7—O4	−156.7 (2)	C6—C5—O1—C14	69.8 (3)
C5—C6—C7—O4	21.8 (3)	O3—C7—O4—C8	−5.3 (4)
C13—C8—C9—O5	179.9 (2)	C6—C7—O4—C8	172.4 (2)
O4—C8—C9—O5	6.0 (3)	C13—C8—O4—C7	105.2 (3)
C13—C8—C9—C10	0.6 (4)	C9—C8—O4—C7	−80.8 (3)
O4—C8—C9—C10	−173.3 (2)	C8—C9—O5—C16	−177.1 (2)
O5—C9—C10—C11	−179.6 (2)	C10—C9—O5—C16	2.2 (4)
C8—C9—C10—C11	−0.4 (4)		
